# Borrowed or kept? How self-generation-first and direct-adoption AI workflows shape immediate unaided reasoning

**DOI:** 10.3389/fpsyg.2026.1910545

**Published:** 2026-09-14

**Authors:** Chenghai Liu, Jiayao Guo, Rongxia Gao, Xiangnan Chen, Qiuhan Zhu, Pengcheng Chang

**Affiliations:** 1 School of Kinesiology and Physical Education, Zhengzhou University, Zhengzhou, Henan, China; 2 Jiangpu Street Community Hospital, Nanjing, Jiangsu, China; 3 School of Management, Zhengzhou University, Zhengzhou, Henan, China

**Keywords:** academic writing, AI workflow design, cognitive offloading, generation effect, generative AI, near transfer

## Abstract

Whether generative AI erodes the skills it assists is usually studied by counting how much people use it, and while the tool is still in hand. That design cannot separate cause from selection, or distinguish using AI from depending on it. We report a three-arm experiment (900 randomized; 659 in the primary analysis) that varies the manner of AI use against a no-AI anchor and measures performance 15–20 min later, once the tool has been removed. Participants wrote an argumentative essay under a self-generation-first autonomous workflow (commit one's own position and then interrogate AI), a direct-adoption dependent workflow, or no AI and then wrote a second, parallel essay with no AI available. Two blind raters scored reasoning quality, idea diversity, and originality. On the unaided task, dependent users reasoned worse than participants who had never used AI (adjusted difference = −0.38 on a 1–7 scale, a small effect, *d* = −0.28, two-sided *p* = 0.0002), whereas autonomous users did not fall below that baseline. The pattern held under per-protocol, covariate-adjusted, Phase-1-time-adjusted, and full-randomized-sample multiple-imputation checks. A trait disposition toward autonomous offloading was discriminant from reflective thinking and critical-thinking disposition (HTMT ≤ 0.43) and incrementally predicted unaided reasoning. We did not detect a metacognitive pathway, although the design cannot exclude one. Because the two workflows differ in self-generation, elaboration, revision, and effort as well as in autonomy, the estimate belongs to the workflow contrast as a whole rather than to any single component: A generation-effect account is consistent with the pattern, but this design cannot weigh it against the alternatives, and no mechanism is established here. The result is an immediate near-transfer decrement rather than demonstrated lasting de-skilling. What separated the arms was not access to the model but the order in which the interaction required the user to produce something.

## 1 Introduction

People have always handed cognitive work to the world around them, storing information in notes, devices, and other people rather than in memory (Risko and Gilbert, 2016; Sparrow et al., 2011). Generative AI extends this habit from storage to production: It has moved from retrieving information to producing the intellectual product itself. A single prompt now returns a structured argument, a counterargument, and a polished paragraph, and field experiments show that this assistance measurably raises the speed and rated quality of written work (Noy and Zhang, 2023). As the tool takes over more of the reasoning rather than merely holding information for later, a practical question follows: When the assistance is withdrawn, what is left in the user? Educators and cognitive scientists increasingly frame this as a risk of de-skilling, in which habitual delegation of thinking may weaken the capability it was meant to support (Dergaa et al., 2024; Jose et al., 2025; Leon-Dominguez, 2024).

The evidence behind this concern is suggestive but cannot yet establish it. Survey and classroom studies report that heavier reliance on AI tools is associated with weaker critical thinking and more cognitive offloading (Gerlich, 2025; Gonsalves, 2026), that over-reliance on dialogue systems correlates with poorer self-reported cognition (Zhai et al., 2024), that students with weaker critical-thinking dispositions rely on AI more readily (Hou et al., 2025), and that learners working with generative AI can fall into what Fan et al. (2025) call metacognitive laziness, offloading the regulation of learning along with its execution. These findings share two limits. They are correlational, so the people who lean on AI may already differ in motivation or skill, and they measure outcomes while the AI is present, so they describe assisted performance rather than what the learner retains alone. Neither limit is incidental: The de-skilling claim is fundamentally causal and about what survives unaided, yet the available evidence speaks to neither.

A separate and older literature manipulates cognitive offloading experimentally but asks a different question. Work in that tradition varies whether or when a person stores information externally and measures the consequences for the task at hand (Risko et al., 2014; Berry et al., 2019; Weis and Wiese, 2019; Grinschgl et al., 2023). Within it, metamemory beliefs govern when people choose to offload (Hu et al., 2019), framing the same act as a gain or a loss shifts how much they rely on it (Froscher et al., 2022), and offloading reshapes the cognitive architecture of the task itself (Skulmowski, 2023). This work establishes that offloading is strategic and that it reshapes concurrent performance, but it treats offloading as a single act that one either performs or does not, and it measures memory rather than the regulation of one's own thinking (Frazier et al., 2021). It does not separate two people who both consult an external aid yet engage with it in opposite ways: one who adopts its output wholesale, and one who treats it as material to be checked, compared, and revised. Learning theory predicts that this difference should matter for what endures. Retention and transfer track the germane cognitive work the learner performs (Sweller, 1988), not the quality or fluency of the material received, so material that one generates oneself is remembered better than material merely read (Slamecka and Graf, 1978; Roediger and Karpicke, 2006), and conditions that feel harder at the time often produce more durable learning (Bjork and Bjork, 2011; Weissgerber et al., 2018; Klingenberg et al., 2020). On this view, the consequence of AI for later competence may turn less on how much help is taken than on how much of the reasoning the user still performs.

Two gaps follow. First, no experiment has manipulated the manner of offloading, how a person uses AI rather than whether they use it, and then measured performance on a later task with the AI removed. Studies of AI-assisted writing have described how students and AI divide the work (Nguyen et al., 2024), tested AI's effect on writing quality and motivation while it is in use (Song and Song, 2023; Niloy et al., 2024), and shown that well-designed scaffolds can support self-regulated learning rather than supplant it (Lim et al., 2023; Chiu, 2024). None has compared an autonomy-preserving against a dependence-inducing workflow and then measured unaided performance, so assisted and retained competence remain confounded and the protective and harmful forms of AI use cannot be distinguished. Second, people may differ stably in how they offload. Recent scale-development work distinguishes dependent from autonomous offloading to generative AI as separable dispositions rather than as two ends of a single reliance dimension (Zhu et al., 2026), but any such disposition has to be placed against the critical-thinking and reflective-thinking traits psychology already measures (Kember et al., 2000; Phan, 2007; Quinn et al., 2020; Bravo et al., 2020). A disposition to interrogate rather than adopt AI output is conceptually close to critical-thinking disposition applied to a tool. We therefore treat it not as an entirely new trait but as a candidate AI-contextualized facet of critical thinking, and ask the narrower, testable question of whether it is empirically separable from, and incrementally predictive beyond, general thinking dispositions.

We address both gaps with a three-arm experiment. Participants wrote an argumentative essay under one of three conditions: an autonomous workflow that required them to form and commit their own position before consulting AI and then to interrogate its output, a dependent workflow that directed them to adopt AI output with minimal change, or no AI at all. They then wrote a second, parallel essay with no AI available to any arm, scored blind, which separates retained competence from concurrent assistance. Anchoring the comparison on the no-AI control lets us ask the strong form of the question: not only whether autonomous use beats dependent use, but whether dependent use leaves people no better off, or worse off, than never having used AI. Generative AI differs from earlier study aids in one respect that matters here: It can return a complete reasoning product, so whether the user still performs the reasoning becomes a feature of how the tool is designed and used rather than a limitation of the learner. Cognitive agency, by which we mean the extent to which reasoning during AI use is performed by the learner rather than delegated to the model, is the idea from which this design grew. We name it to explain where the manipulation came from and then set it aside: It is not measured here, it enters none of the models we estimate, and it carries no inferential weight in what follows. Three qualifications bound every claim below. First, because agency is unmeasured, any statement that it is what carries the effect would be an interpretation rather than a result, and we do not advance it as an explanation of these data. Second, the two AI workflows differ in more than autonomy. The autonomous arm must produce a thesis and outline, compare its own points against the models, adjudicate disagreements, and rewrite; the dependent arm need do almost none of this. Self-generation, elaboration, revision, and effort therefore vary together with manner of use, by design rather than by oversight. A generation-effect account predicts the resulting difference without invoking autonomy at all; an autonomy account predicts it without invoking self-generation. A three-arm design that moves these components together cannot weigh one against the other, so we attribute the effect to the workflow contrast as a whole, name the candidate accounts in the Discussion, and adopt none of them as the explanation. Third, Phase 2 follows Phase 1 by 15–20 min within a single session, so what we can measure is an immediate near-transfer decrement, not the durable erosion that “de-skilling” ordinarily denotes; we reserve that term for the literature's concern and describe our own finding in the narrower language throughout. Figure 1 sets out the design and the contrasts it licenses. We set out a single a priori primary endpoint, the contrast of dependent vs. no-AI on unaided reasoning quality, and test three hypotheses: that the two workflows yield different concurrent, AI-assisted performance (H1), that on the immediately following unaided task dependent users fall below the no-AI baseline while autonomous users do not (H2, the primary contribution), and that a trait disposition toward autonomous offloading is discriminant from and incremental to existing thinking-disposition measures (H3). H1 is a substantive prediction about an assisted outcome, and we no longer describe it as a manipulation check. Whether the manipulation was in fact delivered is established separately, by the behavioral process log (Section 3.1), which records what participants did rather than how well they scored; using a predicted outcome difference as evidence that the manipulation worked would leave that check unfalsifiable. We additionally examine, as an open research question rather than a directional prediction, whether state metacognitive monitoring or intrinsic motivation help account for the transfer effect.

**Figure 1:**
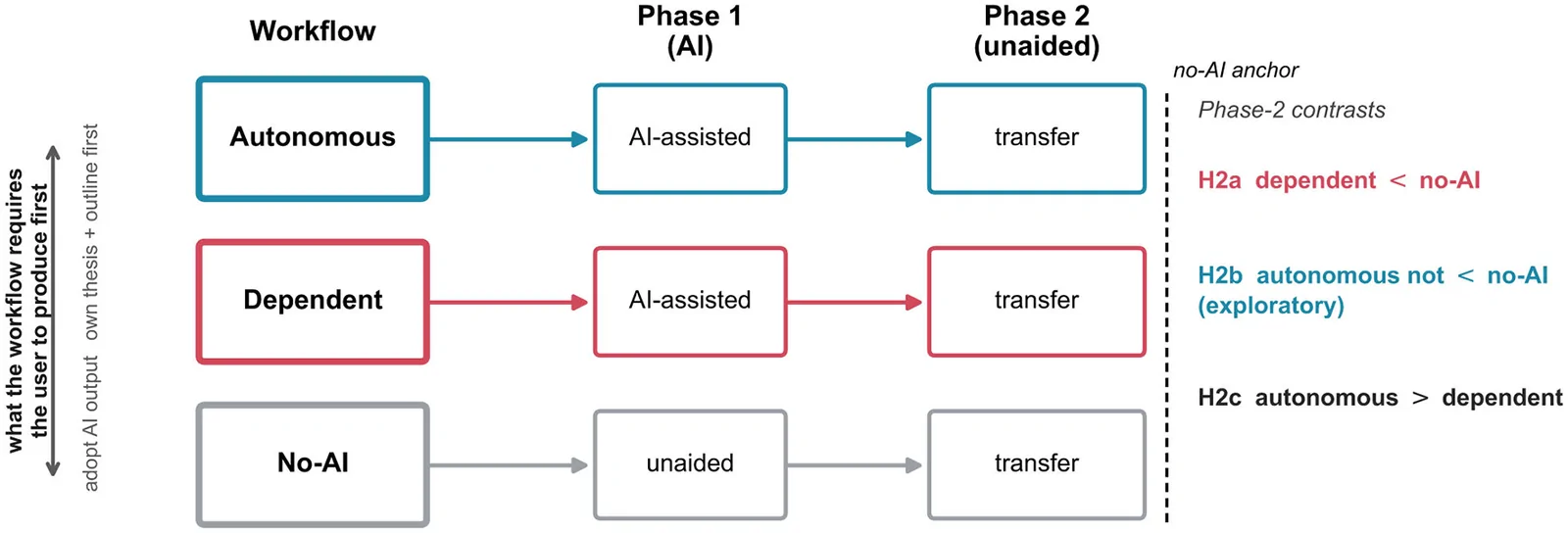
Design and the contrasts it licenses. Cognitive agency does not appear in this figure. It is the concept from which the design grew, but it was not measured, enters none of the estimated models, and is therefore shown neither as a variable nor as a path (Section 4.3). The vertical ordering instead marks a design feature that can be verified from the process log: What each workflow requires the participant to produce before the model produces anything. Participants are randomized to three workflows: autonomous (teal; draft and commit a position first, then interrogate AI), dependent (red; adopt AI output with minimal change), and no-AI (gray). Because the autonomous workflow also entails more elaboration, revision, and effort than the dependent workflow, these components are confounded with autonomy by design, and generation-effect, effort, and autonomy accounts all predict the same pattern; the figure therefore labels the workflow contrast, not an isolated causal ingredient. Each participant completes an AI-assisted Phase-1 task and then an unaided Phase-2 task 15–20 min later, with the no-AI arm as the anchor. The three contrasts listed at right are Phase-2 contrasts against that anchor: H2a (immediate unaided decrement), H2b (protection; exploratory, and not a test of equivalence), and H2c (gradient); they are a list rather than row-wise annotations. H1, which concerns concurrent Phase-1 assisted performance, the behavioral fidelity checks, the trait analysis (H3), and the exploratory mechanism analyses are reported in the text but not depicted. A baseline unaided essay serves as a covariate.

## 2 Materials and methods

### 2.1 Design

The study used a single-factor, three-level between-subjects design (offloading manner: autonomous vs. dependent vs. no-AI) with a within-subjects transfer structure: an AI-assisted task (Phase 1) followed by an unaided parallel task (Phase 2). Phase 2 followed Phase 1 within the same session, roughly 15–20 min later, so the design tests near transfer to an immediately following unaided task, not long-term retention. The study was approved by the Life Science Ethics Committee of Zhengzhou University (Approval No. ZZUIRBSK2025-1932), and all participants gave informed consent before taking part.

#### 2.1.1 What was fixed in advance, and what was not

The study was not formally preregistered, so we state explicitly which decisions preceded the data and which did not, and we report a sensitivity analysis for every decision in the second class. Fixed in a dated internal analysis plan written before the data were analyzed: the three-arm structure and its instructions; the primary endpoint (dependent vs. no-AI on Phase-2 reasoning quality); the direction of H1–H3; the covariate set for each model (baseline ability plus same-phase time on task); the decision not to condition the primary transfer model on Phase-1 performance; the exclusion criteria of failed attention checks, missing Phase-2 data, long-string responding, and indications of AI use in the no-AI arm; the criteria used for that AI screening, which were written down before any essay was scored; and the family-wise treatment of the three objective outcomes under Benjamini–Hochberg correction. Set after the data were in hand, and therefore reported here as exploratory or as sensitivity analyses rather than as confirmatory tests: the 0.3 SD non-inferiority margin, which we now replace with the interval itself (Section 3.4); the per-protocol thresholds (no outline submitted; fewer than half of AI suggestions adopted); and the absence of a numeric lower bound on completion time, which we had planned to set from pilot timings and which no observed case triggered. Section 3.7 reports the primary contrast across the full range of each of these choices; none of them is load-bearing.

### 2.2 Participants

Adults who reported using generative AI for cognitive tasks were recruited through an online research participant platform, with quotas across student and early-career-worker status. Of 900 participants who began the study, we excluded those who failed either attention check (89), did not complete the Phase-2 task (95), showed indications of AI use in the no-AI condition (69; see below), or produced long-string responding (10), leaving a primary analysis sample of *N* = 659 (autonomous = 222, dependent = 225, no-AI = 212; mean age 24.6 years, 54% women). A time-plausibility screen excluded no one; we did not fix a numeric lower bound in advance, and the fastest observed completions (5.0 min for Phase 1, 4.0 min for Phase 2) were within plausible limits. The three arms did not differ on baseline writing ability, age, or self-reported AI-use frequency (all *p*>0.25; Table 1).

**Table 1:** Sample characteristics by condition (primary analysis sample, *N* = 659).

Characteristic	Autonomous (*n* = 222)	Dependent (*n* = 225)	No-AI (*n* = 212)	Test
Age (years)	24.8 (4.7)	24.5 (4.9)	24.5 (4.7)	*F* = 0.38, *p* = 0.69
Women (%)	55	57	50	–
Baseline writing ability	3.86 (1.10)	3.98 (1.13)	3.83 (1.08)	*F* = 1.13, *p* = 0.33
AI-use frequency (1–7)	4.26 (1.31)	4.34 (1.39)	4.14 (1.20)	*F* = 1.37, *p* = 0.26
Phase-1 time (min)	14.2 (2.4)	13.1 (2.7)	13.3 (2.8)	*F* = 11.70, *p* < 0.0001
Phase-2 time (min)	11.3 (2.4)	11.8 (2.4)	11.5 (2.3)	*F* = 2.83, *p* = 0.06

Values are mean (SD) unless noted. The test column reports the one-way ANOVA across the three arms for continuous variables. The first four rows are pre-treatment variables and test randomization balance; the two time-on-task rows are post-treatment and are reported as descriptive evidence about what the manipulation changed, not as balance checks. The autonomous arm spent longer on Phase 1 than the dependent arm (*d* = 0.45); Table 4 shows that adjusting for this does not change the primary contrast.

Because losses of this size can themselves distort a randomized comparison, Table 2 reports the flow by arm and by reason. Randomization produced 278, 294, and 328 assignments to the autonomous, dependent, and no-AI arms; the departure from equal allocation is within chance for a blocked sequence with incomplete final blocks [χ^2^(2) = 4.35, *p* = 0.11]. Exclusion rates differed across arms [20.1%, 23.5%, 35.4%; χ^2^(2) = 20.22, *p* < 0.0001], and the difference is entirely attributable to the AI screen, which by construction can only be applied in the no-AI arm. The three screens that apply identically to all arms did not differ across them: failed attention checks [χ^2^(2) = 0.64, *p* = 0.73], non-completion of Phase 2 [χ^2^(2) = 3.40, *p* = 0.18; and χ^2^(1) = 1.80, *p* = 0.18 between the two AI arms], and long-string responding [χ^2^(2) = 0.80, *p* = 0.67]. Excluded participants had slightly lower baseline ability than retained participants (3.64 vs. 3.89, *p* = 0.003) and reported using AI more often (4.52 vs. 4.25, *p* = 0.009), so exclusion was not independent of participant characteristics; because this holds in every arm and the arms do not differ on the arm-neutral screens, it bears on generalization rather than on the internal comparison. Section 3.6 reports the primary contrast under every relaxation of these exclusions, including an analysis of all 900 randomized participants with missing outcomes imputed.

**Table 2:** Participant flow and exclusions by arm.

Stage or criterion	Autonomous	Dependent	No-AI	Test
Randomized	278	294	328	χ^2^(2) = 4.35, *p* = 0.11
Failed an attention check	29	31	29	χ^2^(2) = 0.64, *p* = 0.73
Did not complete Phase 2	26	39	30	χ^2^(2) = 3.40, *p* = 0.18
Long-string responding	4	2	4	χ^2^(2) = 0.80, *p* = 0.67
Flagged by the AI screen	–	–	69	not applicable
Excluded (any reason)	56	69	116	χ^2^(2) = 20.22, *p* < 0.0001
Exclusion rate (%)	20.1	23.5	35.4	
**Primary analysis sample**	**222**	**225**	**212**	
Per-protocol sample	172	201	212	

Categories are not mutually exclusive; a participant may meet more than one criterion, so the reason counts sum to more than the number excluded. The AI screen applies to the no-AI arm by construction. Tests are χ^2^ across the three arms. Bold values indicate the primary analysis sample (N = 659), on which all main analyses are based.

### 2.3 Procedure

After consent and demographics, participants completed the trait measures (in randomized order) and then a short unaided baseline essay, scored with the same reasoning-quality rubric as the main tasks, to serve as a covariate. They were then randomized to one of three conditions and shown the corresponding instructions and workflow for Phase 1, an AI-assisted argumentative essay of 300–500 words under a 12–15 min limit. After Phase 1, they completed the process measures (manipulation-check items, state metacognitive monitoring, and intrinsic motivation), and then the Phase-2 unaided transfer task, a structurally parallel argumentative essay of 300–500 words under a 10–12 min limit, with no AI available to any arm. A debriefing followed.

The two main topics were public-policy questions of comparable difficulty, deliberately unrelated to AI or technology to avoid confounding with the manipulation: whether private cars should be banned from city centers (Topic A) and whether the standard work week should move from 5 days to 4 days (Topic B). Topic and phase were counterbalanced across participants, so that half wrote Topic A under AI and Topic B unaided and half the reverse, crossed with condition. The baseline essay used a separate policy prompt, given in Section 2.5.

### 2.4 Manipulation

In the autonomous condition, an interface step-lock required participants to draft their own thesis and outline and submit it before AI was unlocked; they were then instructed to compare the AI's points against their own, mark disagreements, judge which were correct, and integrate or rewrite the final text themselves. In the dependent condition, participants were directed to generate the argument with AI and use its output directly, with minimal change, and there was no self-drafting step. In the no-AI condition, participants wrote without any AI or external tools. Both AI conditions drew their AI content from the same model and the same prompt-template pool, so the conditions differed only in how the participant was required to treat that output, not in its source or quality; Section 2.5 documents the system in full. A behavioral log recorded whether an outline was submitted first, the number of AI calls, the proportion of AI suggestions adopted vs. rewritten, and time on task; Section 3.1 reports these as the evidence that the manipulation was delivered. We emphasize that the dependent condition is a deliberate dependence-inducing boundary condition, not a model of typical AI use; it represents one end of a manner-of-use continuum, used here to test whether that end carries a transfer cost.

### 2.5 The AI system

Participants never used a public chatbot interface. All AI content was served through the study platform, which called a single large language model, Qwen3.5 (Tongyi Qianwen; Alibaba Cloud), through its official API. One model version was pinned for the whole data-collection period, and the sampling parameters, temperature, top-*p*, and maximum response length, were fixed at the same values for every participant in both AI arms. A single system prompt, identical in the two AI arms, cast the model as a general-purpose Chinese-language writing assistant; it contained no wording that encouraged either adopting or questioning its output, so the entire autonomous-dependent difference was carried by the front-end interface rather than by anything the model was told. The number of calls per participant was not capped; the observed range was one to eight (median three). The platform logged the full text of every prompt and every response, with timestamps.

The workflows were enforced by interface locks rather than by advice. In the autonomous arm, the AI panel stayed hidden until the participant submitted a written position and a three- to five-point outline of at least 80 characters (“Before using AI, first write down your own position on this issue and an outline of three to five points. At least 80 characters. You can proceed only after submitting.”). The AI panel then opened next to a comparison box in which participants had to set their own outline against the model's points, record at least one disagreement, and judge which side was better founded. A third step required the final text to be organized and rewritten in the participant's own words rather than copied in blocks. In the dependent arm, there was no outline step and a single screen read: “Use AI to complete this argumentative essay. You may have the AI generate the arguments and paragraphs directly; adopt the AI output as it stands, with minimal changes.” Words for comparing, disagreeing, or evaluating never appeared in the dependent instructions. The no-AI arm was told to write independently and was prohibited from using any AI tool, search engine, or external material. Phase 2 used identical wording for all three arms, prohibiting AI and external tools for everyone.

The three writing prompts were fixed in advance: The baseline essay asked whether primary and secondary schools should abolish paper homework in favor of electronic homework (120–200 words, 5–7 min), and Topics A and B are given in the Procedure. The complete Chinese instruction scripts, the system prompt, the prompt-template pool, the pinned model version with its sampling parameters, the three writing prompts, and the rating rubric are available from the corresponding authors upon reasonable request (see Data Availability). One limit on reproducing this study cannot be engineered away: the model is a commercial, closed-weight system that its provider may update without notice, so an investigator issuing the same prompts later may not receive the same text. That is why the call-level record was logged and retained rather than the configuration alone. What participants actually saw is recoverable from those logs even when the model behind it is not.

### 2.6 Outcomes and blind rating

The three objective outcomes, namely, reasoning quality (the primary outcome within each task), idea diversity, and originality, were each scored on a 1–7 rubric with defined anchors. Reasoning quality captured the clarity of the thesis, the relevance and sufficiency of evidence, the coherence of the reasoning, and the handling of counterarguments; idea diversity captured the number and breadth of distinct perspectives; originality captured novelty relative to templated answers. The baseline essay was scored on reasoning quality only. Two raters, blind to both condition and phase, scored essays from which outlines, AI markers, and other metadata had been removed and which had been shuffled. Raters were trained on the rubric with shared exemplars and then calibrated on 10–15 essays until intraclass agreement reached ICC(2,k) ≥0.75 before formal scoring. Each essay was scored by at least two raters; disagreements of two or more points were resolved by a third rater. Rater means were used as the outcome scores.

Responses in the no-AI condition were screened by the research team for textual signatures of AI generation, using criteria fixed before outcome scoring: prose that was implausibly fluent and tightly organized for a timed unaided draft, together with characteristic stylistic markers of chatbot output, such as formulaic connectives and typographic quotation marks and dashes uncommon in hand-typed student writing. The 69 responses flagged on these criteria (21.0% of the no-AI arm and 7.7% of those recruited) were excluded because the no-AI arm is the reference against which both other arms are judged and covert AI use there would compromise it.

We are explicit about the limits of this procedure. The judgment was a single consensus decision by the research team rather than an independent double rating, so we cannot report an inter-rater reliability coefficient for it, and stylistic screening for AI authorship has no established accuracy benchmark. It is, unavoidably, the least objective decision in the pipeline, and it applies to one arm only. Three things bound the damage it can do. First, the flagged responses came from participants who reported using AI substantially more often in daily life (4.65 vs. 4.14 on the 1–7 frequency item, *p* = 0.003, *d* = 0.41) but who did not differ from the rest of the arm in baseline writing ability (*p* = 0.32), age (*p* = 0.75), or trait autonomous offloading (*p* = 0.31), which is the pattern expected if the screen tracked a propensity to reach for AI rather than simply removing the strongest writers. Second, the flagged responses were not in fact higher-scoring than the rest of the arm on the Phase-2 outcome (3.86 vs. 3.81, *p* = 0.73), so retaining or removing them moves the reference mean very little in either direction. Third, and most directly, Section 3.6 adds these participants back and, separately, adds them back in the order most damaging to our conclusion; the primary contrast survives in every configuration. A screening rule that cannot be validated should not be load-bearing, and here it is not.

### 2.7 Trait and process measures

Participants completed the Autonomous Cognitive Offloading (ACO) subscale (6 items) of the Dependent–Autonomous Cognitive Offloading Scale (DACOS; Zhu et al., 2026), the trait disposition of interest. Because that instrument is recent, its evidentiary status should be stated plainly. It was developed and validated in a separate, published three-study program in the same population of students and early-career knowledge workers (Zhu et al., 2026): item generation and expert review; an exploratory sample (*n* = 377) in which parallel analysis and exploratory factor analysis recovered the two-factor dependent/autonomous structure; and an independent confirmatory sample (*n* = 560) in which the two-factor model fit acceptably and the ACO subscale showed α = 0.87, CR = 0.88, AVE = 0.54, and heterotrait–monotrait ratios below the 0.85 threshold against AI dependency, automation complacency, critical AI literacy, and self-regulated learning. That validation is peer-reviewed but recent, and it has not yet been used widely enough to have accumulated independent replication. We therefore re-examined the subscale's properties in the present sample, which is independent of both development samples, and report the result in Section 3.8, including one indicator that falls short of the conventional benchmark; the H3 analyses remain secondary to the experimental findings, and we read them accordingly. As discriminant targets they completed the Reflective Thinking Questionnaire (8 items used; Kember et al., 2000) and the Critical Thinking Disposition Inventory (10 items used; Peng et al., 2004); the reflective-thinking and critical-thinking items were translated and back-translated for this study. Candidate mechanisms were a state metacognitive-monitoring scale (4 items) and the interest (4 items) and effort (4 items) subscales of the Intrinsic Motivation Inventory (McAuley et al., 1989), a self-determination-theory measure (Deci and Ryan, 1985); a single item assessed AI-use frequency. All items showed positive item-rest correlations, so scale scores were unweighted item means.

### 2.8 Randomization and analysis

Participants were assigned by stratified block randomization (strata: baseline-ability tertile crossed with student or worker status; block size six; equal allocation), with the assignment sequence generated and recorded by the online system. All confirmatory tests were analyses of covariance (ANCOVA) with the no-AI arm as the reference category. For Phase-2 (transfer) models, the covariates were baseline writing ability and Phase-2 time on task, and for Phase-1 models, baseline ability and Phase-1 time. We did not enter Phase-1 performance as a covariate in the primary transfer model because it is a post-treatment variable whose inclusion invites Lord's paradox; that model is reported separately as a secondary check. The three objective outcomes were treated as one family, with Benjamini–Hochberg false-discovery-rate (FDR) correction within H1 and within H2. Tests were two-sided at α = 0.05; although H1, H2a, and H2c are directional, we report two-sided *p*-values throughout to be conservative, with 95% confidence intervals for description. A sensitivity analysis confirms the study was well-powered for the primary contrast: The achieved cells (209–225 per arm) detect a between-arm difference of *d* = 0.27 at 80% power (two-sided α = 0.05), and the observed effect exceeds this threshold. The protection hypothesis (H2b) was originally framed as a non-inferiority test against a 0.3 SD margin. Since that margin was chosen once the data were in hand, we no longer report it as a test. We instead report the interval for autonomous minus no-AI and let it speak for itself, and we label the protection conclusion exploratory throughout.

The primary analysis was by assigned condition, but we use the term modified intention-to-treat (mITT) rather than intention-to-treat for it, because it analyzes 659 of 900 randomized participants. Full-sample analyses are reported alongside it in Section 3.6: an available-case analysis of every randomized participant who produced a Phase-2 essay (*n* = 805), a stepwise relaxation of each exclusion rule in turn, a multiple-imputation analysis covering all 900 randomized participants, and a worst-case tipping-point analysis for the non-completers. A per-protocol analysis, with analyst-set thresholds, excluded participants whose logged behavior violated their assigned workflow (autonomous participants who did not submit an outline first; dependent participants who adopted fewer than half of the AI suggestions); we report it as a sensitivity analysis, and a strong conclusion required it to agree with the mITT result. For the multiple-imputation analysis, missing Phase-2 outcomes and covariates were imputed by chained equations under a missing-at-random assumption (m = 20, conditioning on assigned arm, baseline ability, both phase durations, Phase-1 performance, AI-use frequency, age, and the three trait scales), with estimates combined by Rubin's rules.

## 3 Results

### 3.1 The manipulation was delivered (fidelity checks)

Whether participants actually worked the way their arm required is a question about behavior, and the process log answers it without reference to any outcome score. The two AI arms diverged on both locked steps. An acceptable position-and-outline submission preceded AI access for 172 of 222 autonomous participants (77.5%) against 8 of 225 dependent participants [3.6%; χ^2^(1) = 253.9, *p* < 0.0001], the latter having typed an outline into the drafting box unprompted, since the dependent interface contained no outline step. Of the AI content offered to them, autonomous participants adopted a mean of 32% without rewriting (SD = 20) against 73% (SD = 20) in the dependent arm [*t*(445) = −21.38, *p* < 0.0001, *d* = −2.02]. The arms did not differ in a way suggesting unequal access to the model: Autonomous participants made slightly fewer calls (M = 2.72, SD = 1.21) than dependent participants (M = 3.22, SD = 1.56; *d* = −0.36), within the same observed range of one to eight for both.

Two features of these numbers matter. First, they are behavioral and could have come out otherwise: had the step lock failed or the dependent instruction been ignored, the log would show it. Fidelity is high but not complete, and the roughly one in five autonomous participants who never produced an acceptable outline are precisely those removed by the per-protocol analysis (Table 2). Second, and for that reason, we do not treat the Phase-1 performance difference reported next as evidence that the manipulation worked. An outcome difference in the predicted direction cannot play that role, because a null result there would be read as a weak effect rather than as a failed manipulation, which would leave the check unfalsifiable. The check has to rest on what participants did. Self-report manipulation-check items were also administered after Phase 1, and we likewise do not rest the fidelity claim on them: an instructed workflow is precisely the kind of manipulation whose self-report invites demand characteristics, whereas the log records the behavior itself.

### 3.2 Concurrent AI-assisted performance differed between the workflows (H1)

On the AI-assisted Phase-1 task, autonomous users outperformed dependent users on reasoning quality [adjusted difference = +0.78, 95% CI [0.60, 0.96], *p* < 0.0001, *d* = 0.75] and idea diversity (+0.61, *p* < 0.0001), with a smaller advantage on originality (+0.21, *p* = 0.048). All three survived FDR correction. This is a substantive result about assisted performance, not a manipulation check, and it carries one limit worth stating at once: A Phase-1 score in the AI arms rates a human–model product, so it is not directly comparable to the unaided writing produced in the no-AI arm. The workflows also differed on the process indices they were built to differ on: The autonomous arm spent longer on Phase 1 (14.24 vs. 13.11 min, *d* = 0.45), reported more effort on the Intrinsic Motivation Inventory (4.22 vs. 3.91, *p* = 0.015), and reported higher state metacognitive monitoring (4.48 vs. 3.76, *p* < 0.0001). These differences are the confound taken up in Section 4.3: a workflow that requires one group to generate its own reasoning and another to adopt the tool's output moves writing, time, and effort together by construction, which is why the transfer effect cannot be attributed to manner of use in isolation. What none of it reveals, and what the Phase-2 task is built to show, is what remains once the AI is gone.

### 3.3 Measurement reliability

Internal consistency was acceptable for the focal trait scales (ACO α = 0.77, reflective thinking α = 0.81, critical-thinking disposition α = 0.78) and lower for the short mechanism scales [metacognitive monitoring α = 0.65; IMI interest α = 0.62, effort α = 0.58; Table 3]. Blind-rating reliability was high for every outcome [ICC(2,k): Phase-1 reasoning 0.85, diversity 0.84, originality .73; Phase-2 reasoning 0.86, diversity 0.81, originality 0.79], so no outcome was downgraded.

**Table 3:** Descriptive statistics, internal consistency, and inter-construct correlations for the trait and process scales (analysis sample).

Scale	M	SD	ACO	RT	CTD	MM	IM
ACO	4.04	1.36	(0.77)				
RT	4.03	1.30	0.34	(0.81)			
CTD	4.02	1.17	0.32	0.14	(0.78)		
MM	4.04	1.40	0.25	0.13	0.17	(0.65)	
IM	4.00	1.37	0.13	0.02	0.06	0.01	(0.62)

Diagonal entries are Cronbach's α. ACO, autonomous cognitive offloading; RT, reflective thinking; CTD, critical-thinking disposition; MM, state metacognitive monitoring; IM, intrinsic motivation (interest). Heterotrait–monotrait ratios for ACO were 0.43 [bootstrap 95% CI [0.35, 0.51]] with RT and 0.41 [0.33, 0.49] with CTD, both intervals lying entirely below the 0.85 discriminant threshold. In this sample, the ACO subscale also showed CR = 0.78 and AVE = 0.37, the latter below the conventional .50 benchmark (see Section 3.8).

### 3.4 Dependent use is followed by lower unaided performance; autonomous use is not (H2)

The transfer task is where assisted and retained competence diverge, and it carries the study's primary claim. On Phase-2 reasoning quality, with no AI available to anyone, dependent users scored below participants who had never used AI [adjusted difference = −0.38, 95% CI [−0.59, −0.18], *d* = −0.28, two-sided *p* = 0.0002, FDR *q* = 0.0006; Figure 2A]. In this workflow, a single session of dependent AI use was followed, 15–20 min later, by unaided writing that was rated worse than that of a group which wrote unaided throughout. Idea diversity showed the same pattern (−0.30, *d* = −0.23, *p* = 0.004, *q* = 0.006). Originality did not differ (−0.07, *d* = −0.02, *q* = 0.28), so the difference was confined to the reasoning-bearing outcomes rather than to generative novelty. The effect is modest in size (about 0.4 points on a 7-point scale), as expected from a single brief exposure; whether anything like it accumulates under repeated use is a question this design cannot address.

**Figure 2:**
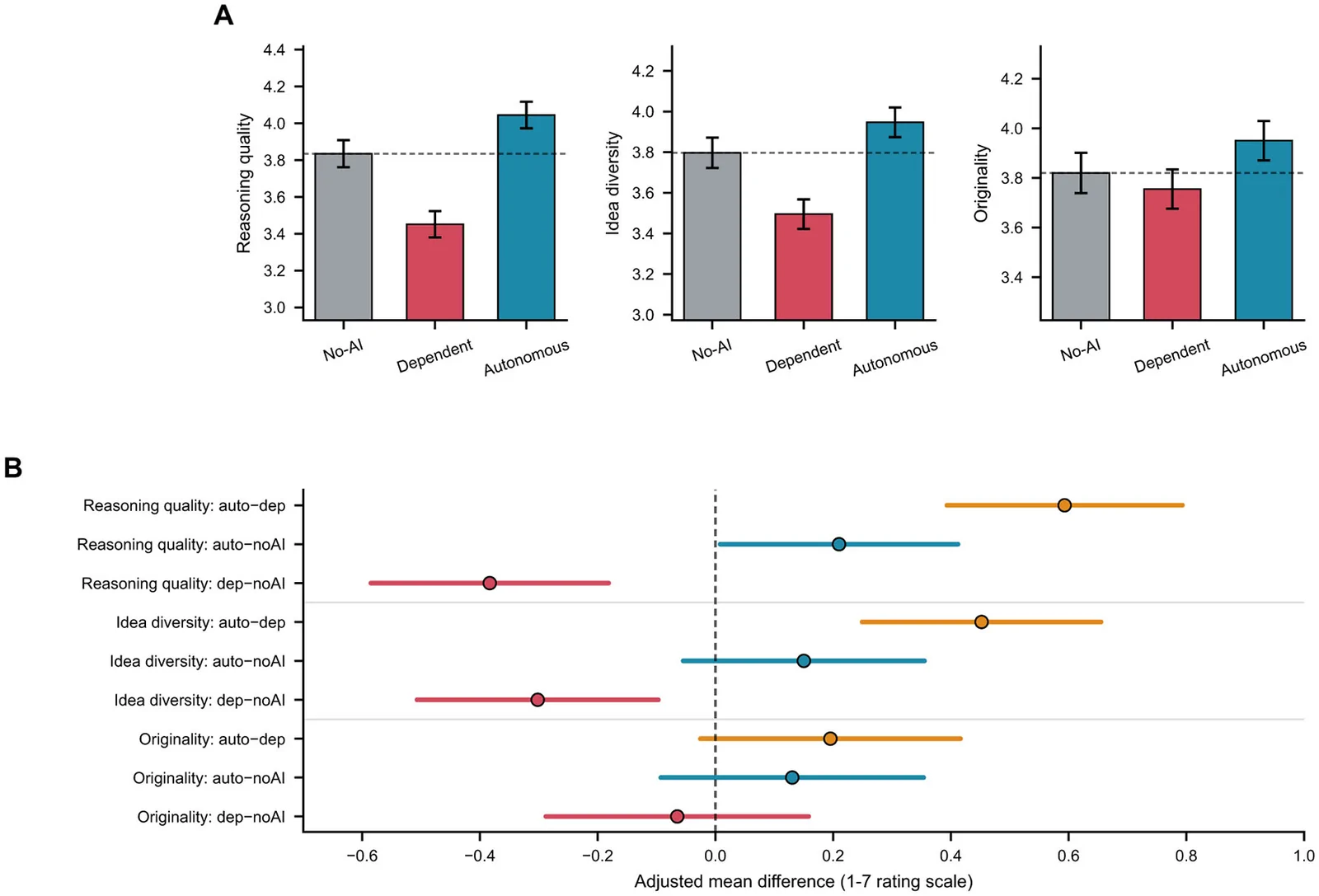
Phase-2 (immediate unaided task) results, primary analysis sample (*N* = 659). **(A)** Covariate-adjusted ANCOVA means by condition for reasoning quality, idea diversity, and originality (error bars ±1 SE; 1–7 blind-rated scale); bars from left to right are no-AI (gray), dependent (red), and autonomous (teal), and the dashed line marks the no-AI baseline. **(B)** Adjusted mean differences with 95% confidence intervals for the three contrasts within each outcome: red, dependent minus no-AI (immediate unaided decrement); teal, autonomous minus no-AI (protection; exploratory); amber, autonomous minus dependent (gradient); the dashed line is the null. For reasoning quality and idea diversity, the dependent–no-AI interval lies entirely below zero and the autonomous–dependent interval entirely above it; all originality intervals span zero. The autonomous–no-AI interval for reasoning quality excludes zero at the 95% level but does not survive correction within the three-outcome family and is not interpreted as a superiority claim.

Autonomous use showed the opposite profile. It exceeded dependent use on reasoning quality by +0.59 (*d* = 0.45, *p* < 0.0001, FDR *q* < 0.0001). Relative to the no-AI baseline, it was +0.21 [95% CI [0.01, 0.41], 90% CI [0.04, 0.38]]. We describe this as a failure to fall below the baseline rather than as a demonstrated advantage. The uncorrected two-sided test is nominally significant (*p* = 0.042), but it does not survive correction within the three-outcome family (*q* = 0.12), and the corresponding contrasts for idea diversity (+0.15, *p* = 0.15) and originality (+0.13, *p* = 0.25) do not approach significance. What can be said without an analyst-chosen margin is that the interval excludes any decrement: The entire 90% confidence interval lies above zero, so the protection conclusion does not depend on where a non-inferiority margin is set. It remains exploratory because the direction of interest was fixed after the fact. Figure 2B shows the full contrast structure: Across reasoning quality and idea diversity, the dependent–no-AI interval lies entirely below zero, the autonomous–dependent interval entirely above it, and the autonomous–no-AI interval brackets or sits just above zero.

### 3.5 The primary contrast is robust to specification (H2, robustness)

The transfer difference held across analytic specifications on this sample; Table 4 collects them. Under the per-protocol sensitivity analysis, it was −0.41 [95% CI [−0.62, −0.20], *p* = 0.0002; *n* = 585]. The ANCOVA assumptions held: The covariate-by-condition interaction was non-significant (slope homogeneity, *F* = 0.29, *p* = 0.75), and residual variance was homogeneous (Breusch–Pagan *p* = 0.26); residuals departed mildly from normality (Shapiro *p* = 0.004), which is immaterial at this sample size and confirmed by heteroscedasticity-robust standard errors (HC3: −0.38, robust *SE* = 0.10, *p* = 0.0002). Substituting either rater's raw scores for the two-rater mean left the contrast in the same range (−0.44 and −0.33, both *p* < 0.004).

**Table 4:** Sensitivity of the primary contrast (dependent minus no-AI on Phase-2 reasoning quality) to specification, sample definition, and analyst-set thresholds.

Specification	Estimate [95% CI]	*p*	*n*
Primary and model specification			
Primary model	−0.38 [−0.59, −0.18]	0.0002	659
Per-protocol	−0.41 [−0.62, −0.20]	0.0002	585
HC3 robust standard errors	−0.38 [−0.59, −0.18]	0.0002	659
Rater 1 scores only	−0.44 [−0.66, −0.23]	0.0001	659
Rater 2 scores only	−0.33 [−0.54, −0.11]	0.0036	659
Adjusting for Phase-1 investment (post-treatment; sensitivity only)			
+ Phase-1 time on task	−0.39 [−0.59, −0.19]	0.0002	659
+ total time across both phases	−0.37 [−0.58, −0.17]	0.0003	659
+ self-reported effort (IMI)	−0.38 [−0.59, −0.18]	0.0002	659
+ Phase-1 time and effort	−0.39 [−0.59, −0.19]	0.0002	659
+ Phase-1 performance (Lord's paradox)	−0.29 [−0.49, −0.10]	0.0032	659
+ all of the above	−0.30 [−0.49, −0.10]	0.0027	659
Sample definition (relaxing each exclusion in turn)			
Retaining attention-check failures	−0.41 [−0.61, −0.22]	< 0.0001	735
Retaining AI-screen-flagged no-AI responses	−0.38 [−0.57, −0.18]	0.0001	713
Retaining long-string responders	−0.38 [−0.58, −0.18]	0.0002	667
All randomized with a Phase-2 essay	−0.41 [−0.59, −0.23]	< 0.0001	805
All randomized, multiple imputation (*m* = 20)	−0.39 [−0.57, −0.21]	< 0.0001	900
Analyst-set thresholds			
Per-protocol adoption threshold 0.3	−0.41 [−0.61, −0.20]	0.0001	602
Per-protocol adoption threshold 0.7	−0.46 [−0.69, −0.23]	0.0001	525
Completion-time floor of 5 min	−0.38 [−0.58, −0.18]	0.0003	656
Completion-time floor of 8 min	−0.38 [−0.59, −0.17]	0.0005	594

All models are ANCOVAs adjusting for baseline writing ability and Phase-2 time on task unless the specification states otherwise; *p*-values are two-sided and uncorrected.

Two of these specifications answer specific concerns about what else the workflows changed. First, the autonomous arm spent about a minute longer on Phase 1 than the dependent arm (14.24 vs. 13.11 min, *t* = 4.72, *p* < 0.0001, *d* = 0.45), so time on task during the manipulation is not equated across arms. Adding Phase-1 duration to the transfer model does not change the picture: Dependent minus no-AI becomes −0.39 [95% CI [−0.59, −0.19], *p* = 0.0002], and autonomous minus dependent becomes +0.63 (*p* < 0.0001), with idea diversity likewise unchanged (−0.30, *q* = 0.006). Adjusting instead for total time across both phases, or for self-reported effort on the Intrinsic Motivation Inventory, or for both together with Phase-1 duration, yields estimates between −0.37 and −0.39 in every case. Phase-1 duration is essentially uncorrelated with unaided Phase-2 reasoning in this sample (*r* = −0.07), which is why controlling it moves nothing. Second, with Phase-1 performance added as a covariate, the secondary model noted above as vulnerable to Lord's paradox, the contrast attenuates but persists (−0.29, *p* = 0.003); because the primary and Phase-1-adjusted estimates point the same way, the transfer difference is not simply a carry-over of lower concurrent performance. We stress that none of these adjustments identifies a mechanism. Duration, effort, and Phase-1 output are all post-treatment variables and all of them shift with the manipulation by design; that they do not account for the effect shows only that a crude time-on-task explanation is insufficient, not that self-generation has been ruled in or out.

### 3.6 Full-sample, attrition, and missing-data analyses

The primary sample is 659 of 900 randomized participants, so we report what happens as that restriction is lifted (Table 4, lower panel). Relaxing each exclusion rule in turn leaves the contrast between −0.38 and −0.41: Retaining participants who failed an attention check gives −0.41 (*p* < 0.0001, *n* = 735); retaining the no-AI responses flagged as possibly AI-written gives −0.38 (*p* = 0.0001, *n* = 713); retaining long-string responders gives −0.38 (*p* = 0.0002, *n* = 667). Analyzing every randomized participant who produced a Phase-2 essay, with no quality screening at all, gives −0.41 [95% CI [−0.59, −0.23], *p* < 0.0001, *n* = 805]. Extending to all 900 randomized participants by multiple imputation of missing outcomes gives −0.39 (95% CI [−0.57, −0.21], *p* < 0.0001). The estimate is therefore not a product of the screening decisions; if anything, the screens make it slightly more conservative.

Two adversarial checks bound the remaining exposure. For the 95 participants who did not complete Phase 2, we replaced the missing outcome with values chosen to work against our hypothesis, assigning dependent-arm non-completers a high quantile of their arm's observed distribution and no-AI non-completers a low quantile. The contrast survives when the assumed gap is moderate (−0.26, *p* = 0.005, at the 60th vs. 40th percentile; −0.22, *p* = 0.020, at the 70th vs. 30th) and loses significance only once one assumes that dependent-arm non-completers would have written at the 80th percentile of their arm while no-AI non-completers wrote at the 20th of theirs (−0.15, *p* = 0.12), an assumption for which there is no support and which runs against the observed direction of the effect. For the AI screening, we added the flagged no-AI participants back in the order most damaging to our conclusion, lowest Phase-2 scores first. The contrast is never weaker than −0.29 at any point along that sequence and remains significant throughout (*p* < 0.005), ending at −0.38 when all are restored; adding them back highest-first strengthens it to −0.47. The conclusion does not turn on this screen in either direction.

### 3.7 Sensitivity to analyst-set thresholds

Three decisions were made after the data arrived, and each was varied across its plausible range. The per-protocol adoption threshold, set at 0.5, gives −0.41; sweeping it from 0.3 to 0.7 gives −0.41, −0.40, −0.41, −0.44, and −0.46, all with *p* ≤ 0.0002, so the more strictly protocol adherence is enforced the larger the estimate becomes. Imposing a completion-time floor, which the original analysis did not, changes nothing: floors of 4, 5, 6, 7, and 8 min on either phase give −0.38 throughout (*p* ≤ 0.0005), losing between 3 and 65 participants. The non-inferiority margin is addressed in Section 3.4: Because the interval for autonomous minus no-AI lies entirely above zero at the 90% level, no margin in the range we considered (0.10 to 0.50 SD) changes the verdict, which is precisely why we now report the interval instead of a test against a margin.

### 3.8 Autonomous offloading is separable from adjacent traits and incrementally predictive (H3)

The trait disposition toward autonomous offloading was empirically separable from the constructs it could be confused with. Heterotrait–monotrait (HTMT) ratios were well below the 0.85 threshold for both reflective thinking [0.43, bootstrap 95% CI [0.35, 0.51]] and critical-thinking disposition [0.41, [0.33, 0.49]; Table 3], with the upper bound of each interval far from the threshold. In hierarchical regressions on the unaided Phase-2 outcomes, controlling AI-use frequency, reflective thinking, and critical-thinking disposition, trait ACO added incremental prediction of reasoning quality (Δ*R*^2^ = 0.017, *p* = 0.0006) and idea diversity (Δ*R*^2^ = 0.010, *p* = 0.008), though not originality.

Since the instrument is new, we also re-examined its psychometric behavior in this independent sample rather than relying on the development study. Internal consistency was acceptable (α = 0.77, CR = 0.78); parallel analysis retained a single factor (first eigenvalue 2.84 against a 95th-percentile criterion of 1.18, second observed eigenvalue 0.88 below its criterion of 1.10), accounting for 47.3% of item variance; and all six item-rest correlations were positive (.32 to .61). Average variance extracted was 0.37, below the conventional 0.50 benchmark and below the 0.54 reported in the validation study (Zhu et al., 2026), driven mainly by one weak item (λ = 0.37; the other five loaded 0.58 to0 .71). The scale also behaved as expected against an external criterion, correlating with unaided Phase-2 reasoning among AI-exposed participants (*r* = 0.16, *p* = 0.0005), though not with the within-session behavioral indices (adoption proportion *r* = 0.09, *p* = 0.053; outline submission *r* = −0.02). We therefore report H3 as showing that the disposition is separable from adjacent traits and modestly predictive, while noting that the convergent-validity evidence in this sample is weaker than in the development study and that a six-item scale with one underperforming item warrants revision before heavier use. The incremental variance is small (approximately 1–2%), as expected when a manipulation already absorbs most of the outcome variance. We therefore read these results as showing that the disposition is empirically separable from, and adds modest prediction beyond, existing measures, but not as establishing a wholly new trait. The more cautious and, we think, more accurate reading is that autonomous offloading behaves as an AI-contextualized facet of critical-thinking disposition; whether it merits status as a distinct construct is a question for dedicated construct-validation work, not for this experiment.

### 3.9 Metacognition and motivation as candidate mechanisms (exploratory)

We examined, as an exploratory question, whether state metacognitive monitoring or intrinsic motivation help account for the dependent-vs.-no-AI effect on reasoning quality. We did not detect either path. Metacognitive monitoring predicted reasoning quality [b-path = +0.16, 95% CI [0.09, 0.23], *p* < 0.001], but the dependent-vs.-no-AI contrast did not move it [a-path = −0.14, 95% CI [−0.40, 0.12], *p* = 0.30], leaving an indirect effect indistinguishable from zero [−0.02, 95% bootstrap CI [−0.07, 0.02]]; the direct effect remained large (−0.36, *p* = 0.0004). The path through intrinsic motivation was likewise undetected.

This is a null result, and its interpretive reach is narrow. Three limits are worth stating rather than glossing. The monitoring scale was four items with α = 0.65, so attenuation is substantial; disattenuating the b-path and pairing it with the observed a-path still bounds the implied indirect effect at roughly 9% of the total effect, but that calculation inherits the imprecision of the a-path. The confidence interval around the indirect effect is compatible with a mediated share of up to approximately 18% of the total effect in either direction. And the a-path has limited resolution: with this sample and this measure, the smallest treatment-to-mediator effect detectable at 90% power is approximately 0.24 SD, so any true path smaller than that would not have been found. What we can say is that a metacognitive route large enough to carry this effect is inconsistent with the data. We cannot say that no metacognitive route exists, and Section 4.3 does not treat this null as evidence for the alternative account.

### 3.10 Process-log indices and unaided performance (exploratory)

Indices drawn from the process log converge with the experimental contrasts. Within the two AI conditions, the more a participant adopted AI output rather than rewriting it, the worse their unaided Phase-2 reasoning (standardized β = −0.21, *p* < 0.001, controlling baseline ability and time), and participants who drafted their own outline first reasoned better unaided (*b* = +0.43, *p* < 0.001). These indices record what the participant did with the AI output, and they are not independent evidence: They are near-deterministic consequences of the assigned workflow, so within-condition residual variation is small and the association is largely carried by the between-condition difference already reported. We report them as convergent description rather than as a mechanism test, and we do not read them as a measure of cognitive agency.

## 4 Discussion

### 4.1 What the experiment establishes

Twenty minutes after a single writing session, participants who had been instructed to adopt AI output reasoned worse without the tool than people who had never used it, while participants who had been required to commit to their own position before consulting AI did not fall below that baseline. Stated at the level the design supports: A self-generation-first AI workflow produced better immediate unaided reasoning than a direct-adoption workflow, and the direct-adoption workflow left users below a no-AI reference. Because the comparison is anchored on a no-AI control and the outcome is measured with the tool absent, this addresses the concern behind the de-skilling literature in a form the prior correlational work could not test: not that AI users underperform while assisted, but that one way of using AI can leave people worse off, on these measures and at this interval, than not using it.

Two words in that literature do not belong to our result, and we drop them. The first is de-skilling. What we measured is a decrement on a structurally parallel task administered 15–20 min later in the same session. That is near transfer within one sitting, and it is compatible with several processes that have nothing to do with skill erosion, among them a carry-over of the shallow processing set induced by the Phase-1 instructions, or a within-session priming of a template the participant had just adopted. Durable erosion of a capability is a claim about repeated exposure over weeks and months, and nothing in a single session speaks to it. The second is dependence as a disposition. The dependent arm did not become dependent; it was instructed, once, to work in a particular way.

### 4.2 Where this moves the debate

Concerns about AI and thinking have been posed largely in terms of quantity: how much people offload (Gerlich, 2025; Zhai et al., 2024), and in the classical experimental tradition, whether and when they offload (Risko et al., 2014; Weis and Wiese, 2019; Grinschgl et al., 2023). Individual-difference work has already shown that dependent and autonomous offloading to generative AI are separable dispositions rather than opposite ends of one reliance dimension (Zhu et al., 2026); what has been missing is an experiment that assigns the two manners of use and then measures what each leaves behind. Our results show that what differed between the arms, and what tracked their later unaided performance, was the structure of the interaction rather than access to the model. The practical corollary is that the step-locks, prompts, and sequencing that determine what a user must produce before the model produces anything are a lever on retained performance, and access to the model is not. This aligns the AI question with a long-standing finding in instruction that guidance helps most when it still requires the learner to do the generative work rather than substituting for it (Kirschner et al., 2006; Zimmerman, 2002).

### 4.3 Mechanism: what the manipulation confounded, and what follows

The two AI workflows differ in more than the manner of AI use. The autonomous arm generated a thesis and outline, compared its own points against the models, adjudicated the disagreements, and rewrote the result; the dependent arm did almost none of this. Self-generation, elaboration, revision, and cognitive effort therefore vary together with autonomy, and the arms also differed measurably in time on task and in self-reported effort. This is a designed feature of the manipulation, not an oversight, but it has a consequence we should state rather than absorb: Several accounts predict our result equally well, and this design separates none of them. A generation-effect account (Slamecka and Graf, 1978; Roediger and Karpicke, 2006; Klingenberg et al., 2020) predicts it with no appeal to autonomy, agency, or dependence, since the arm that produced more of its own reasoning in Phase 1 reasoned better in Phase 2. An effort or time-on-task account predicts it from the fact that the autonomous arm simply worked longer and harder, although the duration and effort adjustments in Section 3.5 tell against the crudest version of that story. An account in terms of autonomy or agency predicts it as well and is the one we can say least about, because neither was measured. We name the generation effect first because it is the most parsimonious of the three and the best established elsewhere, not because these data favor it over the others, and we draw no mechanistic conclusion from that ordering. The contribution we claim is about the workflow contrast, not about the process inside it.

Cognitive agency, the concept from which this design grew, was never measured. The behavioral indices we report, outline submission and the proportion of AI suggestions adopted, are consequences of the assigned workflow rather than independent readings of how much reasoning the user performed, and they cannot license the inference that agency is the operative variable. We therefore make no claim about agency from these data. The term marks the question that motivated the experiment and that a study measuring agency would have to answer; it does no explanatory work here, and it is for this reason absent from Figure 1.

The null result for metacognitive monitoring does not change this. A four-item scale with α = 0.65 and an a-path resolution of roughly 0.24 SD cannot rule out a metacognitive contribution, and we now report the bounds explicitly rather than reading the null as support for the generative account. What the two findings jointly establish is narrower and, we think, still useful: The transfer difference is not explained by a shift in state metacognitive monitoring large enough for this design to see, and it is not explained by time on task, which is uncorrelated with unaided performance here.

Isolating the active ingredient requires designs this study does not contain. A yoked condition that equates total time and effort while varying only who authors the argument would separate self-generation from effort. A condition in which participants generate their own position and then receive a human-written counterargument would separate self-generation from AI as such. A direct process measure of generative engagement, rather than a behavioral proxy determined by the instructions, would let agency be tested instead of assumed. Until then, the defensible claim is about workflows, not about the mechanism inside them.

### 4.4 External validity and what it means for classrooms

The dependent condition is an instructed extreme. Participants were told to adopt AI output with minimal change, which is not how most people use these tools; real use sits somewhere on a continuum between wholesale adoption and full independence, and it varies within a single task as a writer alternates between drafting and querying. Our estimate is therefore the cost at one boundary of that continuum, deliberately chosen to make the boundary visible, and it should not be read as an estimate for typical use. The autonomous condition is equally artificial in the opposite direction: an interface lock compelled a sequence that few users would impose on themselves. Both arms are instruments for locating the ends of a range, and the magnitude of the effect at the ends says little about its magnitude in the middle, which may be smaller, or absent, or non-linear in ways only a dose–response design could reveal.

What does carry over to practice is narrower than a warning about AI. If the cost concentrates at the adoption end of the continuum, then the target of intervention is the sequence of the interaction rather than access to the tool, and the sequence is something instructors and tool designers already control. Requiring a committed position before the model is unlocked, asking students to mark where they disagree with its output, or grading the revision rather than the product are all instances of the same move: requiring the learner to produce something before the model does. The literature on AI scaffolds points the same way, showing that well-designed supports can sustain self-regulated learning rather than displace it (Lim et al., 2023; Chiu, 2024), that students and AI divide the work in describable ways (Nguyen et al., 2024), and that learners can offload the regulation of learning along with its execution (Fan et al., 2025). Our contribution to that practical literature is a controlled comparison with a no-AI anchor, which shows that the sequencing choice can determine whether an AI-supported session leaves the learner ahead of or behind where they would have been without the tool. Whether the specific step-lock we used is the right classroom intervention is a separate question, and a field study with realistic tasks, repeated exposure, and delayed testing would be needed to answer it.

### 4.5 Limitations

Beyond the mechanistic and external-validity limits already discussed, five constraints bound what can be concluded.

First, the control arm wrote an original essay in Phase 1 while the dependent arm largely did not, so the no-AI arm entered Phase 2 with a rehearsal the dependent arm lacked. This warm-up account is a genuine alternative to any interpretation in terms of what AI use did, and our design cannot exclude it, because the same feature that makes the no-AI arm the right causal anchor also gives it the extra practice. Two observations bear on it without settling it. Relative to the pre-treatment baseline essay, which all arms wrote unaided, the no-AI arm was essentially unchanged (−0.02 on the 1–7 scale), the autonomous arm gained slightly (+0.17), and the dependent arm lost substantially (−0.49; between-arm difference in change scores *F*(2, 656) = 15.66, *p* < 0.0001); a pure warm-up advantage should have raised the no-AI arm above the autonomous arm, which required its own generation but under AI-assisted conditions, and it did not. Phase-1 duration, the crudest index of how much practice each arm accumulated, is uncorrelated with Phase-2 performance and does not alter the contrast when controlled. A fourth arm performing an unrelated filler task in Phase 1 would separate rehearsal from workflow, and we regard it as the most informative single addition to this design.

Second, the primary analysis retains 659 of 900 randomized participants, so it is a modified rather than a full intention-to-treat analysis. The arm-neutral screens did not differ across conditions, the AI screen applies to one arm by construction, and Section 3.6 shows the estimate is stable across every relaxation, including multiple imputation over all randomized participants and a worst-case assignment for non-completers. The screening of the no-AI arm for AI authorship remains the weakest link: It was a single team judgment without an independent second rating, and no accuracy benchmark exists for such judgments. We have shown that it is not load-bearing, but a future study should either double-rate such judgments with a reported reliability coefficient or, better, remove the need for them through supervised administration.

Third, the effect is specific to the reasoning-bearing outcomes and absent for originality, it rests on a single AI model and prompt pool, two policy topics, and one participant population of Chinese-speaking students and early-career workers recruited online, and Phase-1 scores in the AI arms reflect a human–model product that is not directly comparable to the no-AI arm's unaided writing.

Fourth, the protection conclusion for the autonomous arm is exploratory. The direction of the comparison and, originally, the margin were settled after the data were in hand; what survives without an analyst-chosen threshold is that the interval excludes a decrement, not that autonomous use confers a benefit.

Fifth, the trait analyses draw on a recently published instrument (Zhu et al., 2026) whose average variance extracted in this sample fell below the conventional benchmark and which has not yet been replicated by groups other than its developers. They are secondary to the experimental findings and should be read as preliminary.

### 4.6 A note on how the question should be posed

What the data support, more than any single estimate, is a shift in how the question is asked. Whether generative AI dulls the abilities it assists is unlikely to be settled by counting how often people reach for it, because the same amount of use can be organized so as to require very different amounts of work from the user, depending on the order in which the interaction makes things happen. Our experiment demonstrates that two such orders produce different unaided performance immediately afterward. It does not establish which cognitive process makes the difference, nor that anything about it persists. Those are the next two questions, and they need designs that equate effort and follow learners over time.

## 5 Conclusion

In a three-arm experiment, a direct-adoption AI workflow was followed, 15–20 min later, by lower unaided reasoning than never using AI at all, whereas a self-generation-first workflow was not. The estimate was stable across per-protocol, time-adjusted, Phase-1-adjusted, full-sample, and multiple-imputation specifications and under robust standard errors, and a trait disposition toward autonomous offloading predicted unaided reasoning beyond existing thinking-disposition measures, albeit modestly and with an instrument still under development. Because the workflows differ in self-generation, elaboration, revision, and effort as well as in autonomy, the estimate belongs to the workflow contrast as a whole rather than to any component of it; a generation-effect account fits the pattern, but so do accounts in terms of effort or autonomy, and separating them requires a yoked design this study does not provide. What the finding supports is a claim about immediate unaided performance after one session, not about durable skill erosion, and about instructed extremes of a continuum rather than about typical use. Within those bounds, it constrains practice in a specific way: Giving people access to AI does not by itself determine what they can do without it, but the sequence the interaction imposes may.

## Data Availability

The datasets analyzed for this study are not publicly available because they contain participant-level survey responses. De-identified data and analysis materials are available from the corresponding authors upon reasonable request, subject to institutional ethics requirements.
